# Nuvaxovid NVX-CoV2373 vaccine safety profile: real-world data evidence after 100,000 doses, Australia, 2022 to 2023

**DOI:** 10.2807/1560-7917.ES.2024.29.50.2400164

**Published:** 2024-12-12

**Authors:** Hazel J Clothier, Claire Parker, John H Mallard, Paul Effler, Lauren Bloomfield, Dale Carcione, Jim P Buttery

**Affiliations:** 1 Epi-Informatics, Centre for Health Analytics, Melbourne Children’s Campus, Melbourne, Australia; 2 Epi-Informatics Group and SAEFVIC Epidemiology, Surveillance and Signal Detection, Murdoch Children’s Research Institute, Melbourne, Australia; 3 Department of Paediatrics, University of Melbourne, Melbourne, Australia; 4 Melbourne School of Population and Global Health, University of Melbourne, Melbourne, Australia; 5 Communicable Disease Control Directorate, Western Australia Department of Health, Perth, Australia; 6 Immunisation Service, Perth Children’s Hospital, Nedlands, Australia; 7 Infectious Diseases Unit, Royal Children's Hospital Melbourne, Melbourne, Australia

**Keywords:** Vaccine safety, adverse events, surveillance, immunisation, COVID, Nuvaxovid, myocarditis, pericarditis

## Abstract

**Introduction:**

Nuvaxovid became available in Australia from February 2022, a year after the first COVID-19 vaccines. This protein-based vaccine was an alternative for people who had had an adverse event to and/or were hesitant to receive an mRNA or adenovirus-based COVID-19 vaccine. Although safety from clinical trials was reassuring, small trial populations, low administration rates and limited post-licensure intelligence meant potential rare adverse events were underinformed.

**Aim:**

We aimed to describe Nuvaxovid’s safety profile in a real-world setting.

**Methods:**

We conducted a retrospective observational analysis of adverse events following immunisation (AEFI) spontaneously reported to SAFEVAC, the integrated vaccine safety surveillance system in Victoria and Western Australia. Reports from 14 February 2022 to 30 June 2023 were analysed by vaccinee demographics, reported reactions and COVID-19 vaccine dose, and compared as reporting rates (RR) per 100,000 doses administered.

**Results:**

We received 356 AEFI reports, following 102,946 Nuvaxovid doses administered. Rates were higher after dose 1 than dose 2 (rate ratio: 1.5, p = 0.0008), primary series than booster (rate ratio: 2.4, p < 0.0001), and in females vs males (rate ratio: 1.4, p = 0.004). Clinically confirmed serious AEFI included 94 cases of chest pain (RR = 91.3), two myocarditis (RR = 1.9) and 20 pericarditis (RR = 19.4). Guillain–Barré syndrome or thrombosis with thrombocytopaenia syndromes were not reported, nor deaths attributable to vaccination.

**Conclusion:**

SAFEVAC’s collaborative data model enabled pooling of clinically reviewed data across jurisdictions, increasing the safety profile evidence for Nuvaxovid and improving the odds for identification and description of rare events. This analysis affirmed the safety profile of Nuvaxovid.

**Key public health message**

**What did you want to address in this study and why?**

Nuvaxovid is a protein-based vaccine against COVID-19 that can be given as an alternative to adenoviral vector and mRNA COVID-19 vaccines. It received provisional licensure following clinical trials in which 30,058 participants received at least one dose of Nuvaxovid. We wanted to inform on the safety of Nuvaxovid following over 100,000 doses administered in real-world population-wide setting in two Australian states.

**What have we learnt from this study?**

Adverse events reported were mostly mild, transient symptoms, and no deaths were attributed to Nuvaxovid immunisation. The frequency of heart inflammation was similar to the mRNA COVID-19 vaccines, but more likely to be milder pericarditis rather than myocarditis. For over 25,000 people, Nuvaxovid was their first dose of a COVID-19 vaccine received, although it became available more than a year after the other COVID-19 vaccines.

**What are the implications of your findings for public health?**

Our analysis of Nuvaxovid vaccine safety provides favourable benefit–risk profile of vaccination for protection from severe COVID-19 disease. We must continually monitor safety using real-world data, to not only ensure vaccines are safe, but also support confidence in immunisation.

## Introduction

Nuvaxovid (NVX-CoV2373, Novavax) is a protein-based COVID-19 vaccine for prevention of coronavirus disease 2019 (COVID-19) caused by SARS-CoV-2, provisionally approved for emergency use by the World Health Organization (WHO) in December 2021 [1], ahead of full registration by the Australian Therapeutic Goods Administration (TGA) in October 2023 [2]. While protein sub-unit vaccines have been well established for decades, the Matrix-M adjuvant is less familiar, having been used mainly in Ebola vaccines, administered predominantly in low-income countries.

Australia was one of the first countries to use Nuvaxovid in the community, with vaccines administered from mid-February 2022 for primary series in persons aged 18 years and older and extended in July 2022 to individuals from 12 years-of-age and as a booster for those over 18 years [2].

Nuvaxovid was introduced almost exactly 12 months after the adenovirus (Vaxzevria (AstraZeneca) 8 March 2021) and mRNA (Comirnaty (Pfizer) 21 February 2021 and later Spikevax (Moderna) 27 September 2021) vaccines. At the time, over 95% of the eligible population had already received at least one COVID-19 vaccine dose [3]. However, Nuvaxovid provided the only available alternate vaccine for people who had either suffered an adverse event to and/or were unwilling to receive one of the mRNA- or adenovirus-based COVID-19 vaccines [4-7]. A United States (US) survey of 400 individuals hesitant to receive any available COVID-19 vaccines in early 2022 revealed that 55% would probably agree to receive a protein-based vaccine if made available [8].

Provisional registration with the Australian regulatory agency Therapeutic Goods Administration (TGA) was granted in January 2022 on the basis of short-term efficacy and safety data from pooled clinical trial data from ca 50,000 persons ≥ 18 years of whom 30,058 received at least one dose of Nuvaxovid, and a paediatric expansion study of 1,487 adolescents (12–17 years of age) who had received at least one dose [9-11]. Therefore, the TGA determined that “*Continued approval depends on the evidence of longer-term efficacy and safety from ongoing clinical trials and post-market assessment*” [12]. Of particular note were certain adverse events of clinical special interest (AESI) reported infrequently in the clinical trials, but with small numerical imbalances between the Nuvaxovid and placebo arms [13]. These included biliary, neurovascular and cardiac events (including myocarditis) and uveitis [10,13]. In addition, one case of Guillain–Barré syndrome was considered likely to be associated with the vaccine [14].

Our epidemiological analysis of Nuvaxovid safety profile aimed to provide insights into adverse events following immunisation (AEFI), including frequency, severity and sex distribution following over 100,000 doses of Nuvaxovid administered in a real-world setting.

## Methods

### SAFEVAC surveillance system

SAFEVAC is the integrated vaccine safety surveillance platform used by the states of Victoria (VIC) and Western Australia (WA), Australia [15,16], covering an estimated 9,163,517 population (VIC: 6,503,491; WA: 2,660,026) [17]. Reports to SAFEVAC can be made by healthcare providers, vaccinees (or their guardians) or ascertained from integrated surveillance identifying medically attended potential AEFI reported via active surveillance post-vaccination surveys or linked hospital immunisation datasets (WA) [18]. Reporting of adverse events is voluntary in VIC and a statutory requirement for health professionals in WA [19].

Serious AEFI are defined as events that are life-threatening, require in-patient hospital admission or prolongation of admission intervention to prevent injury, or result in death, persistent or significant disability/incapacity, a congenital anomaly/birth defect [20]. SAFEVAC accepts all AEFI reports submitted, therefore it is important to acknowledge the adverse events as temporally associated with immunisation and not assessed as causal [16].

### Clinical review

All reports received via SAFEVAC are triaged by a clinical team of immunisation nurses, with potential serious AEFI or adverse events of special interest (AESI) triaged for confirmation of the clinical details and follow-up if required. Described symptoms and signs are coded as descriptive reaction terms, consistent with medical terminology and/or case definitions as appropriate [15]. All reports are included in the database as being an AEFI temporally associated with vaccination, without requirement for medical record review or causality assessment. However, as myocarditis and pericarditis were of particular interest in relation to COVID-19 vaccines, all reports of these side effects underwent clinical review of reported symptoms, with patient follow-up where contact details were available and consent to contact provided, and categorised according to Brighton Collaboration case definition level of certainty [21].

### Statistical analysis

Adverse events following Nuvaxovid (original COVID-19 vaccine) reported to SAFEVAC from 14 February 2022 to 30 June 2023 were analysed by vaccinee demographics, including reported sex and 10-year age group, reactions, and dose of COVID-19 vaccine received.

We calculated reporting rates per 100,000 doses recorded for VIC and WA for the same period in the Australian Immunisation Register (AIR) by 28 August 2023 [22]. We referred to vaccine dose numbers as primary series (doses 1 and 2) and boosters (dose 3 or more).

Data were visualised using Microsoft Power BI (Microsoft desktop version 2.118.1063.0) and statistical calculations conducted using STATA version 18 (Statacorp) for Poisson 95% confidence intervals (CI), reporting rates (RR) and rate ratios, with Fisher’s exact test used for group comparisons. A p value lower than 0.05 was considered statistically significant. As data on COVID-19 diagnoses were not collected in SAFEVAC, we did not analyse adverse events considering any history of prior or coincident COVID-19 infection.

## Results

### Doses administered

**Figure 1:**
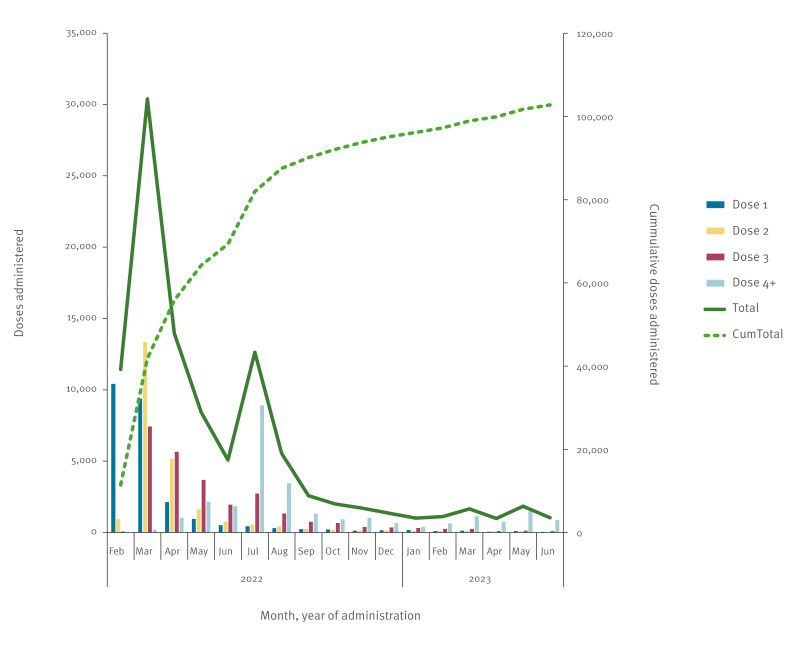
Count of Nuvaxovid doses administered, by dose and month and cumulative total, Victoria and Western Australia, Australia, 14 February 2022–30 June 2023 (n = 102,946)

A total of 102,946 Nuvaxovid doses were administered during the study period (14 February 2022 to 30 June 2023) (54,566 VIC, 48,380 WA). Overall, 48.3% of doses were primary series (dose 1 = 25,496; dose 2 = 24,183; boosters = 53,095), noting this proportion was as high as 74.2% (41,398/55,804) in the first 3 months of the Nuvaxovid roll-out (Figure 1). Distribution by recorded sex was ca 20% higher for females (n = 56,098) than males (n = 46,654) (ratio: 1.2). The AIR data were missing for age (n = 108), sex (n = 194) and dose number (n = 172).

CumTotal: cumulative total.

### Reporting of adverse events following immunisation

A total of 356 AEFI reports (143 VIC, 213 WA) were received, an overall AEFI reporting rate of 345.8 per 100,000 doses. Of these, 123 (34.6%) met the definition of serious AEFI (Table 1).

**Table 1:** Characteristics of adverse event reports to SAFEVAC after Nuvaxovid vaccination, Victoria and Western Australia, Australia, 14 February 2022–30 June 2023 (n = 356)

Nuvaxovid		Dose 1	Dose 2	Primary^a^	Boosters	Total
Doses^b^		25,496	24,183	49,679	53,095	102,946^b^
AEFI reports						
Total	n	153	94	247	109	356
	Rate^c^	600.1	388.7	497.2	205.3	345.8
Serious^d^	n	54	32	86	37	123
	%	35.3	34.0	34.8	33.9	34.6
	Rate	211.8	132.3	173.1	69.7	119.5
Non-serious	n	99	62	161	72	233
	%	64.7	66.0	65.2	65.1	65.4
	Rate	388.3	256.4	324.1	135.6	226.3
Reporter type						
HCW^e^	n	68	49	117	45	162
	%	44.4	52.1	47.4	41.3	45.5
Consumer	n	80	40	120	36	156
	%	52.3	42.6	48.6	33.0	43.8
Other	n	5	5	10	28	38
	%	3.3	5.3	4.0	23.9	10.7
Spontaneous	n	126	76	202	62	264
	%	82.4	80.9	81.8	56.9	74.2
Active^f^	n	27	18	45	47	92
	%	17.6	19.1	18.2	43.1	25.8

AEFI: adverse events following immunisation; HCW: healthcare worker.

^a^ Primary series is dose 1 and dose 2 combined.

^b^ Dose number not known for 172 doses administered.

^c^ Rate per 100,000 doses administered.

^d^ Serious AEFI as defined by World Health Organization definition detailed in methods.

^e^ HCW includes pharmacists.

^f^ Active = solicited medically attended AEFI or active search of hospital administration data.

### Dose

Reporting rate was higher following dose 1 (600.1) than dose 2 (388.7), a rate ratio of 1.5 (95% CI: 1.2–2.0; p = 0.0008). It was higher after the primary series (497.2) than after booster doses (205.3), a rate ratio of 2.4 (95% CI: 1.9–3.1; p < 0.0001). The proportion of reports deemed serious was similar across all doses.

### Age and sex

**Figure 2:**
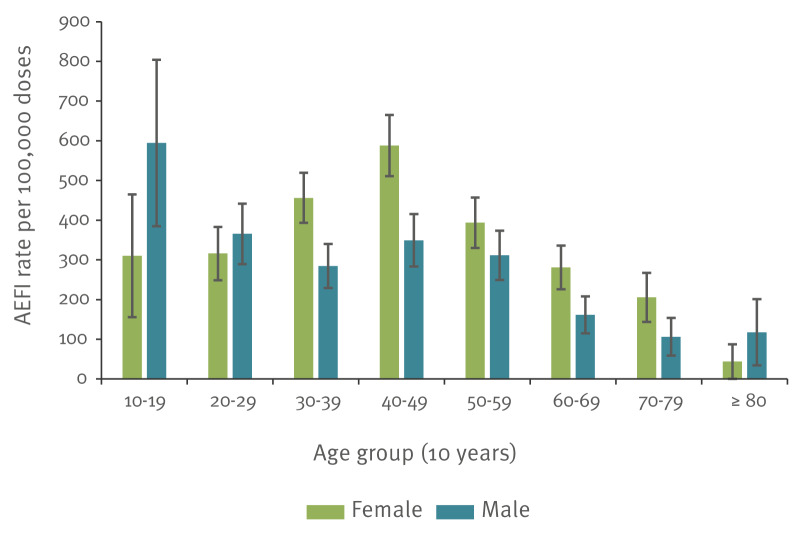
Adverse event following immunisation reporting rate by sex and 10-year age group, with 95% confidence intervals, Victoria and Western Australia, Australia, date (total reports = 356)

We received AEFI reports for persons aged 6–97 years (interquartile range (IQR): 22); age was unknown for nine cases. More reports were for females by count (female = 219, male = 133, neither = 1, not stated = 3) and by rate (rate ratio: 1.4; p = 0.004) (Table 2). Reporting rate varied by age group and sex: higher in females 30–39 and 40–49 years of age (p = 0.047 and p = 0.021, respectively) and in younger males aged 10–19 years, but without reaching statistical significance (p = 0.298) (Figure 2).

**Table 2:** Adverse event following immunisation reactions reported after Nuvaxovid vaccination, as count and rate per 100,000 doses, by sex, Victoria and Western Australia, 14 February 2022–30 June 2023 (total reactions n = 937)

Reaction	Count^a^			Rate per 100,000 doses			F:M rate ratio	p value
	Female	Male	Total^b^	Female	Male	Total		
Doses administered	56,098	46,654	102,946^c^					
Total reports	219	133	356^b^	390.4	285.1	345.8	**1.4**	**0.004**
Chest pain	47	46	94	83.8	98.6	91.3	0.8	0.434
Headache	32	26	58	57.0	55.7	56.3	1.0	0.934
Fatigue or lethargy	33	25	58	58.8	53.6	56.3	1.1	0.730
Myalgia (muscle pain)	32	14	47	57.0	30.0	45.7	**1.9**	**0.041**
Injection site reaction (incl pain)	31	13	44	55.3	27.9	42.7	**2.0**	**0.034**
Paraesthesia (pins and needles)	31	7	38	55.3	15.0	36.9	**3.7**	**0.0006**
Shortness of breath	21	14	35	37.4	30.0	34.0	1.2	0.529
Palpitations	21	13	34	37.4	27.9	33.0	1.3	0.409
Arthralgia (joint pain)	17	11	29	30.3	23.6	28.2	1.3	0.526
Fever	20	6	27	35.7	12.9	26.2	**2.8**	**0.021**
Nausea	22	5	27	39.2	10.7	26.2	**3.7**	**0.004**
Dizziness	13	13	26	23.2	27.9	25.3	0.8	0.642
Myocarditis and pericarditis^d^	12	13	25	21.4	27.9	24.3	0.8	0.508
Cardiac symptoms (incl tachycardia)	5	13	18	8.9	27.9	17.5	0.3	0.253
Rash	14	4	18	25.0	8.6	17.5	**2.9**	**0.049**
Malaise	12	5	17	21.4	10.7	16.5	2.0	0.195
Vaccine error^e^	5	11	16	8.9	23.6	15.5	0.4	0.068
Abdominal pain	11	2	14	19.6	4.3	13.6	**4.6**	**0.030**
Diarrhoea	9	4	13	16.0	8.6	12.6	1.9	0.634
Vomiting	11	2	13	19.6	4.3	12.6	**4.6**	**0.030**
Lymphadenopathy	8	5	13	14.3	10.7	12.6	1.3	0.634
Tachycardia	5	13	18	8.9	27.9	17.5	0.3	0.253
Urticaria/hives	9	3	12	16.0	6.4	11.7	2.5	0.168
Pruritus	8	2	11	14.3	4.3	10.7	3.3	0.117
Influenza-like illness	7	2	9	12.5	4.3	8.7	2.9	0.180
Tinnitus	7	1	9	12.5	2.1	8.7	6.0	0.0068
Anxiety	6	2	8	10.7	4.3	7.8	2.5	0.274
Brain fog	6	2	8	10.7	4.3	7.8	2.5	0.274
Anaphylaxis	7	0	7	12.5	0.0	6.8	NR	**0.014**
Menstrual changes	7	0	7	12.5	0.0	6.8	NR	NR
Pain (other)	4	3	7	7.1	6.4	6.8	1.1	0.908
Generalised allergic reaction	4	2	6	7.1	4.3	5.8	1.7	0.592
Chills	3	3	6	5.3	6.4	5.8	0.8	0.829
Angioedema	4	2	6	7.1	4.3	5.8	1.7	0.592
Migraine	4	2	6	7.1	4.3	5.8	1.7	0.592
Hypertension	3	2	5	5.3	4.3	4.9	1.3	0.836
Insomnia	3	2	5	5.3	4.3	4.9	1.3	0.836
Altered throat sensation	3	2	5	5.3	4.3	4.9	1.3	0.836
Other^a^	94	41	138	167.6	87.9	134.1	**1.9**	**0.0004**
**Total**	**591**	**336**	**937**	**1,053.5**	**720.2**	**910.2**	**1.5**	**< 0.0001**

F: female; M: male; NR: not relevant.

^a^ Reactions with total count < 5 not listed to protect privacy.

^b^ Includes one reaction with sex = neither. For nine reactions (three cases), sex was not stated.

*^c^* Total Australian Immunisation Register dose count includes 194 for whom sex was not recorded.

^d^ Includes three pericarditis cases where an alternate cause was deemed likely.

^e^ Vaccine administration errors did not result in an adverse event following immunisation reaction.

A report may describe > 1 reaction. Figures in bold indicate reactions with statistically significant rate difference by sex.

AEFI: Adverse events following immunisation.

^a^ Data for two cases aged < 10 years not shown (one male, one female).

### Adverse events following immunisation reactions reported

**Figure 3:**
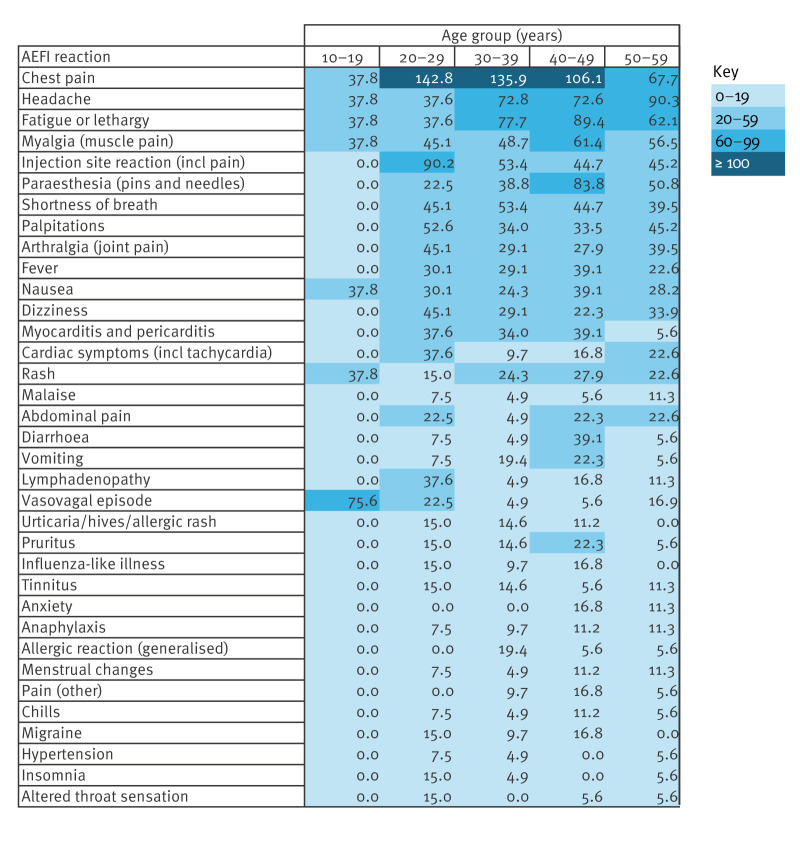
Adverse event following immunisation reactions reported after Nuvaxovid vaccination as rate per 100,000 doses, Victoria and Western Australia, 14 February 2022–30 June 2023 (total reactions n = 937)

In the 356 reports, 937 reactions were described. All reactions reported a total of five or more times are listed in Table 2 by count, sex, rate per 100,000 and female-to-male reporting rate ratio, and in Figure 3 as reporting rate by 10-year age group. The median time to onset was 1 day (range: 0–56 days; IQR: 3.96 for all doses, and range: 0–21 days; IQR: 2.92 following dose 1).

Only reactions reported at least five times are included in the Table. Age unknown for 29 reports.

No cases of facial palsy, Guillain–Barré syndrome (GBS) or thrombosis with thrombocytopaenia syndrome (TTS) were reported. Four deaths identified through active search of hospital datasets were identified as occurring temporally proximal to vaccination, however, investigation determined that no deaths were attributable to vaccination.

Pericarditis was reported after primary and booster doses (total: n = 20; RR = 19.5; 95% CI: 11.9–30.1). Reporting rate was highest following dose 1 (n = 10; RR = 39.2; 95% CI: 18.8–72.1), but similar after dose 2 (n = 3; RR = 12.4; 95% CI: 2.6–36.3) and after boosters (n = 7; RR = 13.2; 95% CI: 5.3–27.2). The two clinically confirmed myocarditis cases (Brighton Collaboration level 1) both occurred following dose 2 (overall RR = 1.95; 95% CI: 0.1–7.0 or RR = 8.3; 95% CI: 1.0–29.9 specifically for dose 2), with onset within 14 days of vaccination in one male and one female, and no history of myocarditis associated with a prior COVID vaccination.

There was one report of uveitis reported on a day 42 active survey response, however, insufficient details meant that we were unable to follow-up for clinical review and confirmation, including days to onset after the third dose of Nuvaxovid was received.

## Discussion

Our study provides a southern hemisphere post-authorisation safety profile of Nuvaxovid, describing adverse events reported following more than 100,000 doses of Nuvaxovid (original SARS-CoV-2 rS vaccine) administered in real-world setting of a high-income country, using both spontaneous and active surveillance for AEFI identification.

Commonly reported AEFI were consistent with clinical trial data in that “*injection site reactions, fatigue, myalgia, headache, malaise, arthralgia, nausea, or vomiting*” all featured in the top 10 reported reactions and that local and systemic adverse reactions occurred more commonly after dose 1 than after dose 2 [10,11]. However, chest pain was the most commonly reported reaction, and shortness of breath and palpitations also featured in the top 10. While the propensity to report these reactions was probably influenced by the acknowledged and broadly communicated risk of myocarditis or pericarditis following mRNA COVID-19 vaccines [23], cardiac disorders were nonetheless noted as new AEFI associated with Nuvaxovid and included in the Nuvaxovid product information [11,24].

We demonstrated slightly higher overall AEFI reporting for females, particularly those aged 30–49 years of age. Sex disproportionality was expected in observed rates of injection site reaction, paraesthesia, nausea and vomiting, consistent with pre-licensure studies [25,26]. However, we also identified disproportional reporting in females for anaphylaxis, which has not been previously reported for Nuvaxovid, although a similar disproportionality had been noted by Somiya et al. in association with mRNA vaccines [27]. The high reporting rate of menstrual changes remained of interest as they had not been noted in clinical trial data. It is not known if this was because of zero reporting, or because trials were not structured to seek information on menstrual changes [26,28]. Increased awareness of menstrual changes associated with COVID-19 vaccines may also have influenced reporting behaviours for these conditions [29].

The reporting of heart inflammation after receiving Nuvaxovid had a distribution pattern consistent with that seen for mRNA COVID-19 vaccines, with risk of myocarditis associated with dose 2 and younger age groups, but pericarditis more likely to be reported following dose 1 and across any age [30]. The reporting rate for myocarditis following Nuvaxovid was lower than for mRNA vaccines but that for pericarditis was higher [30]. Our data are not able to distinguish if this was a shift in clinical manifestation or a bias because people seeking to receive Nuvaxovid were more predisposed to—or aware of the need to report—heart inflammation symptoms.

Our findings corroborate the clinical trial results and the single published post-licensure study we identified, which was from Korea in > 18-year-olds (14 February to 31 December 2022) with 1,230 AEFI reported via spontaneous and active (text message service on days 0 and 7) surveillance following 926,982 doses administered [31]. However, the Korean study reported an overall lower AEFI reporting rate of 132.7 per 100,000 and a lower proportion (7.8%) of serious AEFI. They reported four suspected cases of myocarditis, but no confirmation of cases was described. Korean authors also published a case report of a 30-year-old male with clinically confirmed myocarditis onset 17 days post dose 2 Nuvaxovid as a vaccination complication [32].

In our study, two clinically confirmed cases of myocarditis resulted in a higher overall rate (1.93 per 100,000) than that seen in Korea of (0.11 per 100,000). Cases were consistent with Brighton Collaboration definite level 1 of certainty, but no causality assessment was performed other than noting onset within 14 days of vaccination and the absence of an alternate cause.

The shared SAFEVAC platform allows collaborative pooling of clinically reviewed data across jurisdictions, increasing the evidence base for informing the safety profile of novel vaccines such as Nuvaxovid and improving the ability to detect and describe rare events in all vaccines and/or inform on AEFI in small population groups. In addition, access to robust exposure data via the AIR makes more insights possible than reported AEFI data alone.

Both states (WA and VIC) clinically reviewed reports potentially indicating an AESI, where medical attention was sought, and all myocarditis and pericarditis reports. This enhancement over routine spontaneous surveillance provides insights to inform the level of certainty of diagnoses and any subsequent causality assessment, which cannot be informed by reporting alone. Ability to conduct clinical review of reported reactions of myocarditis was important, as the media advocacy related to mRNA vaccine-associated myocarditis caused an increased propensity to seek medical attention when experiencing any cardiac symptoms such as chest pain [33].

This study was based on enhanced spontaneous surveillance data, which will incur the known biases of spontaneous reporting systems, including under-reporting [16]. Although AEFI reporting is not mandated in VIC, this does not historically result in lower reporting, as is evidenced by VIC consistently being the lead jurisdiction by AEFI reporting volume per population size nationally [16,34]. However, overall AEFI reporting in this study was lower in the more populated state of VIC than in WA, although a similar number of doses was administered in both states. We hypothesise that the stringent pragmatic surveillance modifications introduced in VIC to ensure that routine systems were not overwhelmed by the COVID-19 pandemic created higher proportional reporting of medically attended events such as chest pain [34,35].

This study aimed to describe AEFI reported following vaccination with Nuvaxovid and was not structured to provide a direct comparison with other vaccines administered in different phases of the COVID-19 pandemic or when different surveillance processes were initiated to accommodate the high volume of reporting during the pandemic [34,36]. SAFEVAC collates reports of adverse events and does not have data linkage to disease notification datasets necessary to understand the impact of prior or coincident COVID-19 on AEFI occurrence and/or on an increase in incidence above background rates [37,38]. Nuvaxovid was the only vaccine introduced in Australia during a period of high circulation of COVID-19 [36], therefore caution is advised in making direct comparisons of AEFI incidence between vaccine brands without considering the different periods of the pandemic and of surveillance strategies [39].

Several AEFI showed wide disparity in reporting rate between females and males, notably tinnitus with a rate ratio of 6.0 (p = 0.068), and warrant a close watch. A limitation of early analysis or low numbers of administered is insufficient sample size to inform statistical difference in disproportionate reporting. Thus, collaborative data networks, such as SAFEVAC, that can pool data or conduct meta-analyses can be beneficial when events are rare or when stratified analysis of less common events is required [37].

Some people were cautious about the novel mRNA vaccine types or the rare occurrence of blood clots and cardiac inflammation attributed to specific COVID-19 vaccine brands, and therefore waited for the release of Nuvaxovid [4-8]. Our data confirm this, as more than 74% of all Nuvaxovid doses administered in the first 3 months after its release were part of a primary series, although it was introduced nearly 12 months after the adenoviral vector and mRNA vaccines. This corresponds to more than 25,000 people receiving at least one dose of COVID-19 vaccine who otherwise may not have been vaccinated and indicates not only the importance of enabling choice, but that there is appetite for nuanced vaccine safety profiling.

Our findings provide broad population-level safety intelligence for Nuvaxovid, however, there is increasing demand for personalised information or precision immunisation, and rightly so, for true informed consent for immunisation should be based on individual vaccination risk–benefit. Exploration of linked datasets, or primary care datasets of vaccine administered by general practitioners, could assist in ascertaining not only trends in health outcomes but also if the population receiving Nuvaxovid had a differing co-morbidity profile and/or COVID-19 infection status and/or had cardiac inflammation after a prior COVID-19 vaccination [40] and/or variation in the propensity to seek medical attention, for example for cardiac symptoms.

More broadly, further studies are needed to explore the impact of sex-disproportional AEFI on individual perception of vaccine safety, confidence to vaccinate and whether communication—for example that transient menstrual changes can be anticipated [41]—would allay concerns and increase vaccine acceptance [42,43].

Using the statistical estimation rule of three [44], having 100,000 administered doses approximates 95% confidence of detecting rare AEFI with incidence of more than one in 33,333 vaccinations, far less than the 3 million doses required to detect the very rare one in a million event, or to detect a doubling increase above expected background rates of a rare events [35]. Therefore, it is important to maintain continued vaccine vigilance, particularly as multi-heterologous COVID-19 vaccine exposures and new-variant booster vaccine programmes are now in use.

## Conclusion

Despite its later arrival in Australia’s COVID-19 vaccination programme, Nuvaxovid played a vital role by providing vaccine choice, increasing uptake with potentially 25,000 additional persons accepting a COVID-19 vaccination dose. SAFEVAC’s collaborative data model supporting multi-jurisdictional analysis made clinically reviewed adverse event information available in sufficient volume for meaningful insights. The findings provided reassurance that while pericarditis and myocarditis occurred with similar rates overall as after receipt of an mRNA vaccine, the rates were skewed towards pericarditis, with minimal reports of the more severe myocarditis condition. Robust post-licensure vaccine vigilance using real-world data is essential, especially when aspiring to protect global biosecurity with rapidly deployed new vaccines, not only to ensure safe immunisation, but also to maximise community confidence—as only a vaccine that gets administered can save lives. This epidemiological analysis affirms the safety profile and positive benefit-risk ratio of Nuvaxovid in a real-world setting.
